# Quantifying Repetitive Transmission at Chemical Synapses: A Generative-Model Approach^1^,^2^,^3^

**DOI:** 10.1523/ENEURO.0113-15.2016

**Published:** 2016-05-13

**Authors:** Alessandro Barri, Yun Wang, David Hansel, Gianluigi Mongillo

**Affiliations:** 1 Unité d'Imagerie Dynamique du Neurone, Institut Pasteur, Paris, France; 2 Caritas St. Elizabeth's Center, Tufts University, Boston, MA, USA; 3 Centre National de la Recherche Scientifique, UMR 8119, Paris, France; 4 Cerebral Dynamics, Plasticity and Learning, Centre de Neurophysique, Physiologie et Pathologie, Université Descartes, Paris, France

**Keywords:** expectation-maximization, generative modeling, quantal analysis, repetitive transmission, short-term plasticity

## Abstract

The dependence of the synaptic responses on the history of activation and their large variability are both distinctive features of repetitive transmission at chemical synapses. Quantitative investigations have mostly focused on trial-averaged responses to characterize dynamic aspects of the transmission—thus disregarding variability—or on the fluctuations of the responses in steady conditions to characterize variability—thus disregarding dynamics. We present a statistically principled framework to quantify the dynamics of the probability distribution of synaptic responses under arbitrary patterns of activation. This is achieved by constructing a generative model of repetitive transmission, which includes an explicit description of the sources of stochasticity present in the process. The underlying parameters are then selected via an expectation-maximization algorithm that is exact for a large class of models of synaptic transmission, so as to maximize the likelihood of the observed responses. The method exploits the information contained in the correlation between responses to produce highly accurate estimates of both quantal and dynamic parameters from the same recordings. The method also provides important conceptual and technical advances over existing state-of-the-art techniques. In particular, the repetition of the same stimulation in identical conditions becomes unnecessary. This paves the way to the design of optimal protocols to estimate synaptic parameters, to the quantitative comparison of synaptic models over benchmark datasets, and, most importantly, to the study of repetitive transmission under physiologically relevant patterns of synaptic activation.

## Significance Statement

Transmission at chemical synapses is transiently adjusted on a spike-by-spike basis, which has been proposed to enhance information processing in neuronal networks. So far, however, the dynamic properties of transmission have been characterized only for physiologically unrealistic patterns of activation. This is because the current methods used to estimate the parameters describing repetitive transmission are unable to deal with the fluctuations of the responses. These must either be averaged out or estimated directly from the data, which requires a large number of repetitions of the same stimulation, severely constraining experimental protocols. We developed a novel method that allows one to estimate the parameters from a single, arbitrary pattern of activation. The method lays the groundwork for the characterization of transmission with *in vivo*-like patterns of activation.

## Introduction

A distinctive feature of chemical transmission is the rapid and transient modification of the postsynaptic response as a result of repetitive presynaptic activation (Zucker and Regehr, 2002; Fioravante and Regehr, 2011). The ability of chemical synapses to quickly adjust their transmission properties in an activity-dependent way has been suggested to significantly enhance information processing in neuronal networks. Important computations are thought to rely, fully or partly, on “synaptic computations” (Abbott and Regehr, 2004; Wu et al., 2013). A nonexhaustive list includes the following: rhythm generation (Senn et al., 1996; Tsodyks et al., 2000), gain control (Abbott et al., 1997; Rothman et al., 2009), temporal filtering (Fortune and Rose, 2001), temporary memory maintenance (Hempel et al., 2000; Barak and Tsodyks, 2007; Mongillo et al., 2008), and the source of nonlinearity in the balanced regime (Mongillo et al., 2012; Hansel and Mato, 2013).

Quantitative investigation of repetitive synaptic transmission largely relies on phenomenological descriptions (Bertram et al., 1996; Tsodyks and Markram, 1997; Varela et al., 1997; Markram et al., 1998; Dittman et al., 2000). In their providing compact (i.e., with few parameters), low-dimensional descriptions, phenomenological models have been instrumental in effectively classifying patterns of transmission at different synapses (Gupta et al., 2000; Blackman et al., 2013), in uncovering their underlying mechanisms (Dittman et al., 2000; Saviane and Silver, 2006; Hallermann et al., 2010b), and in exploring theoretically their functional consequences. Phenomenological models, however, only describe the average responses or, where the model is stochastic, it is the average model responses that are fitted to the trial-averaged experimental responses. In either case, the trial-to-trial variability and the within-trial correlation of the responses are neglected (but, see Costa et al., 2013). The variability of the synaptic responses is, indeed, another distinctive feature of chemical transmission. This variability is understood and routinely quantified in terms of the quantal model of synaptic release (Quastel, 1997; Stevens, 2003). Methods to estimate quantal parameters are, however, tailored for steady-state conditions, and their extension to dynamic conditions has proven difficult (Scheuss et al., 2002; Saviane and Silver, 2006; Loebel et al., 2009; Hallermann et al., 2010a,b).

The trial-averaging procedure required to fit models to data destroys the large amount of information contained in the correlation between consecutive responses as well as in their fluctuations. The accuracy of the parameters estimate, achievable by least-squares fitting, is thus seriously limited and steadily declines with increasing the complexity of the model (i.e., with the number of parameters to be fitted). Trial averaging also severely constrains experimental protocols. The need to have a suitable number of repetitions in identical conditions leads, essentially, to protocols consisting of short, regular presynaptic trains at relatively high rates, followed by quite long interstimulation intervals (compared with stimulation periods). The repetition of identical trains (regular or not) allows one to extract very little information about the underlying synaptic dynamics. Moreover, the parameters are estimated with patterns of synaptic activation that are arguably very far from physiological patterns, raising the question of how good a description are the current models and/or parameters for repetitive synaptic transmission in *in vivo*-like conditions (Dobrunz and Stevens, 1999; Kandaswamy et al., 2010).

Here, we provide a new methodology that integrates information about the stochasticity of the synaptic responses in the estimation of the parameters describing the dynamic properties of synaptic transmission. We introduce a novel class of generative models that allows one to compactly describe the statistical dependencies among consecutive synaptic responses as well as their variability. We show that both inference and learning are tractable in this class of generative models. In particular, we develop an exact expectation-maximization (EM) algorithm that allows one to estimate the parameters for arbitrary patterns of presynaptic stimulation. We demonstrate two main advantages of our approach over conventional techniques. First, we simultaneously estimate both quantal and dynamic parameters from the same experimental recordings. Second, and most importantly, since the estimation procedure does not rely on trial-averaged quantities, parameters can be estimated from single traces. It is thus possible to devise alternative stimulation protocols and analyze their impact on parameter estimation by the use of theoretical tools.

## Materials and Methods

### Electrophysiological recordings

We refer the reader to Wang et al. (2006) for a detailed description. Briefly, acute slices were cut from the medial prefrontal cortex of young ferrets (1.5–3 months old), and whole-cell patch-clamp recordings were made from synaptically connected layer 5 pyramidal neurons. Synaptic transmission was probed by eliciting in the presynaptic cell regular trains of five or eight spikes at varying frequencies, followed by a recovery spike. Postsynaptic responses were recorded in current-clamp mode. The interspike interval of the train *T* ranged between 14.3 and 200 ms (5–70 Hz). The interval for the recovery spike was correspondingly determined as *T*_rec_ = *T* + 500 ms. This stimulation protocol was repeated between 20 and 40 times.

Most of the connections were probed with a single frequency of stimulation (20 Hz, i.e., T = 50 ms). For the connections probed with several frequencies, we retained only one for further analysis. The retained frequency was chosen so as to have the largest possible number of connections for each of the frequencies used in the experiment.

### Preprocessing

Postsynaptic voltage traces that exhibited drift or an abrupt change in the baseline during recording were excluded from further analysis. The remaining voltage traces were smoothed by using a rectangular window 2 ms in size. From the smoothed traces, we extracted the single peak responses using the method described by Richardson and Silberberg (2008). Briefly, from the trial-averaged trace we estimated the membrane time constant *τ_m_* by fitting an exponential decay to the falling edge of the recovery response and, where possible, also to the falling edge of the first response, and averaged over these. Each voltage trace *V*(*t*) was then deconvolved using the following:

(1) $$RI(t)=\tau_{m}\cdot \frac{dV(t)}{dt}+V(t).$$

From the *RI*(*t*) trace, which typically featured clearly separated peaks, we cropped 12 ms windows centered around the nominal time of the presynaptic stimulation. These “crops” were then reconvolved, yielding fully separated excitatory postsynaptic potentials, from which we finally extracted the peak responses. From the “crops” we also estimated the baseline noise $\sigma_{n}^{2}$ by computing the variance of the voltage trace over a 1 ms time window before the onset of the response. The baseline noise was estimated separately for each connection.

### The stochastic Tsodyks-Markram model

The stochastic Tsodyks-Markram (TM) model (Fuhrmann et al., 2002) describes the synapse as a collection of *N* identical and independent release sites. Each site can be in one of the two following states: competent (i.e., occupied by one vesicle), or noncompetent (i.e., no vesicle is docked to the site). Upon spike, a competent site can probabilistically release the vesicle, and become noncompetent. The probability of release *u*(*t*) depends on the history of synaptic activation according to the following:

(2) $$\overset{˙}{u}=\frac{U-u}{\tau_{F}}+U\;\cdot \;(1-u)\underset{k}{\sum }\delta (t-t_{k}),$$

where *U* is the initial release probability, *τ_F_* is the time constant of facilitation, and the sum over *k* is over all presynaptic spike times, *t_k_*. In between spikes, vesicles dock probabilistically to noncompetent sites. The probability of docking within a time interval Δ since the site first became noncompetent, *l*(Δ), is given by the following:

(3) $$l(\Delta )=1-e^{-\Delta /\tau_{D}},$$

where *τ_D_* is the average time a vesicle takes to dock to a noncompetent site. The response *R* to one vesicle is a random variable with mean *q* (quantal size) and variance $\sigma_{q}^{2}$ (quantal variability). To account for the right-skewness of unitary quantal responses (Bekkers et al., 1990; Bhumbra and Beato, 2013), we use an inverse Gaussian for the probability distribution function of *R*, as follows:

(4) $$P(R)=\frac{q^{3/2}}{\sqrt{2\pi \sigma_{q}^{2}R^{3}}}\exp[-\frac{q{\left(R-q\right)}^{2}}{2\sigma_{q}^{2}R}].$$

The response to more than one vesicle is the linear sum of single quantal responses (see Eq. 11). The average postsynaptic responses ${\bar{R}}_{k}$ elicited by presynaptic spikes at times *t_k_* are given by the following:

(5) $${\overset{¯}{R}}_{k}=A\;\cdot \;u_{k}\;\cdot \;x_{k},$$

where A≡N·q is the so-called absolute synaptic efficacy, *u_k_* is the probability of release immediately before spike at time *t_k_*, and *x_k_* is the probability that a site is release competent immediately before the spike at time *t_k_*. Both *u_k_* and *x_k_* can be recursively evaluated as follows:

(6) $$u_{k+1}=U+u_{k}\cdot (1-U)\cdot \exp\left(-\frac{\Delta_{k}}{\tau_{F}}\right)$$

(7) $$x_{k+1}=1-\left[1-(1-u_{k})\cdot x_{k}\right]\cdot \exp\left(-\frac{\Delta_{k}}{\tau_{D}}\right),$$

where $\Delta_{k}\equiv t_{k+1}-t_{k}$ is the *k*th interspike interval, *u*_1_ = *U* and *x*_1_ = 1.

### Likelihood of a response sequence

The likelihood of observing a sequence of postsynaptic responses $R_{1\to M}\equiv \left\{R_{1},\cdots \;,R_{M}\right\}$ elicited by a train of presynaptic spikes occurring at times $t_{1\to M}\equiv \left\{t_{1},\cdots \;,t_{M}\right\}$ is given by the following:

(8) $$L(\theta |R_{1\to M})\equiv P(R_{1\to M}|\theta )=\underset{S_{1\to M}^{-},S_{1\to M}^{+}}{\sum }P(R_{1\to M},S_{1\to M}^{-},S_{1\to M}^{+}|\theta ),$$

where $S_{1\to M}^{-}$ and $S_{1\to M}^{+}$ denote the sequences of a number of release-competent sites immediately before and after each spike, respectively. The model parameters are denoted by θ. For concreteness, in the case of the stochastic TM model they are as follows: the number of release sites *N*, the quantal size *q*, the quantal noise $\sigma_{q}^{2}$, the initial release probability *U*, and the time constants for docking and facilitation *τ_D_* and *τ_F_*, respectively. To simplify the notation, we omit hereafter the dependence on the parameters θ assumed to be constant in what follows. The joint probability of $R_{1\to M},\;S_{1\to M}^{-}$ and $S_{1\to M}^{+}$ can be written as follows:

(9) $$P(R_{1\to M},S_{1\to M}^{-},S_{1\to M}^{+})=P(S_{1}^{-})\prod_{k=1}^{M}P(S_{k}^{+}|S_{k}^{-})P(R_{k}|S_{k}^{+},S_{k}^{-})\prod_{k=1}^{M-1}P(S_{k+1}^{-}|S_{k}^{+}),$$

where $P(S_{1}^{-})$ is the probability that $S_{1}^{-}$ sites are competent at the beginning of the spike train. For the stochastic TM model, all sites are release competent in the absence of stimulation. We have $P(S_{1}^{-})=1$ if $S_{1}^{-}=N$ and $P(S_{1}^{-})=0$ otherwise. The term $P(S_{k}^{+}|S_{k}^{-})$ describes the release process (i.e., it is the probability that the number of release-competent sites changes from $S_{k}^{-}$ to $S_{k}^{+}$ upon the *k*th spike). Before the *k*th spike, there are $S_{k}^{-}$ release-competent sites that can independently release with probability *u_k_*. The number of release-competent sites cannot increase upon spike. Thus:

(10) $$P(S_{k}^{+}|S_{k}^{-})=\{\begin{array}{cc} \left(\begin{array}{c} S_{k}^{-}\\ S_{k}^{-}-S_{k}^{+} \end{array}\right)\cdot u_{k}^{\left(S_{k}^{-}-S_{k}^{+}\right)}\cdot {\left(1-u_{k}\right)}^{S_{k}^{+}} & \text{if}\;\;\;\;S_{k}^{+}\leq S_{k}^{-}\\ 0 & \text{otherwise} \end{array}\;.\;\;$$

The term $P(R_{k}|S_{k}^{+},S_{k}^{-})$ describes the postsynaptic responses (i.e., it is the probability of observing a response between *R_k_* and *R_k_* + *dR* when the number of release-competent sites changes from $S_{k}^{-}$ to $S_{k}^{+}$ upon the *k*th spike). Note that $S_{k}^{-}-S_{k}^{+}$ is the number of vesicles released. Because of linear summation, *R_k_* is also distributed according to an inverse Gaussian distribution with mean $(S_{k}^{-}-S_{k}^{+})\cdot q$ and variance $(S_{k}^{-}-S_{k}^{+})\cdot \sigma_{q}^{2}$, as follows:(11)
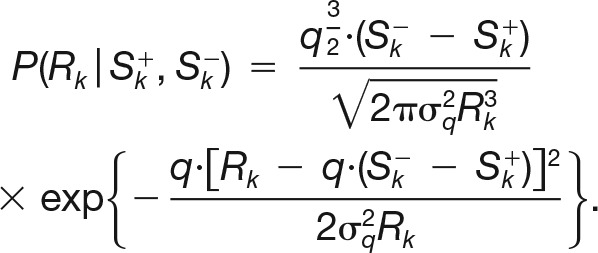



Finally, the term $P(S_{k+1}^{-}|S_{k}^{+})$ describes the docking process, i.e., it is the probability that the number of release-competent sites changes from $S_{k}^{+}$ to $S_{k+1}^{-}$ during the *k*th interspike interval Δ*_k_*. After the *k*th spike, there are $N-S_{k}^{+}$ noncompetent sites that can independently become release competent within the time interval Δ*_k_* with the probability $l_{k}\equiv l(\Delta_{k})$ (see Eq. 3). The number of release-competent sites cannot decrease in between spikes. Thus:

(12) $$P(S_{k+1}^{-}|S_{k}^{+})=\{\begin{array}{cc} \left(\begin{array}{c} N-S_{k}^{+}\\ S_{k+1}^{-}-S_{k}^{+} \end{array}\right)\cdot l_{k}^{\left(S_{k+1}^{-}-S_{k}^{+}\right)}\cdot {\left(1-l_{k}\right)}^{\left(N-S_{k+1}^{-}\right)} & \text{if}\;\;\;\;S_{k+1}^{-}\geq S_{k}^{+}\\ 0 & \text{otherwise} \end{array}.$$

The sum over all possible realizations of $S_{1\to M}^{-}$ and $S_{1\to M}^{+}$ in Equation 8 can be efficiently performed as described in the Forward-backward formalism section.

### Expectation-Maximization

The fact that the number of release-competent sites is not directly observable makes the direct maximization of the likelihood function in Equation 8 impractical. The EM algorithm allows for maximum-likelihood estimation in the presence of hidden variables (Dempster et al., 1977; Dayan and Abbott, 2001). EM iteratively improves upon an initial guess of the parameters by maximizing the so-called auxiliary function with respect to θ. The auxiliary function is defined as follows:

(13) $$Q(\theta ,\theta^{old})=\underset{S_{1\to M}^{-}}{\sum }\underset{S_{1\to M}^{+}}{\sum }P(S_{1\to M}^{-},S_{1\to M}^{+}|R_{1\to M},\theta^{old})\times \log\left[P(R_{1\to M},S_{1\to M}^{-},S_{1\to M}^{+}|\theta )\right],$$

where $\theta^{old}$ is the initial guess for the parameters, and the sum is over all possible sequences $S_{1\to M}^{-}$ and $S_{1\to M}^{+}$. The EM algorithm comprises two steps. The so-called E step corresponds to the evaluation of the auxiliary function, that is, the computation of the expectation of $\log\left[P(R_{1\to M},S_{1\to M}^{-},S_{1\to M}^{+}|\theta )\right]$ given the observed responses and the current estimate. The so-called M step corresponds to maximizing the auxiliary function with respect to θ, as follows:

(14) $$\theta^{new}=\underset{{}_{\theta }}{\text{arg}\;\text{max}\;}Q(\theta ,\theta^{old}).$$

Iteration of the E and M steps guarantees improvement of the initial guess until eventually a fixed point is reached (i.e., $\theta^{new}=\theta^{old}$), which corresponds to a (local) maximum of the likelihood function.

Using Equations 9–12 in Equation 13, taking the derivatives with respect to the continuous parameters, and setting them to 0, we obtain the following re-estimation formulas:

(15) $$\sum_{k=1}^{M}〈R_{k}-q^{new}\cdot (S_{k}^{-}-S_{k}^{+})〉=0[q^{new}].$$

(16) $$\sum_{k=1}^{M}〈1-\frac{q^{new}}{{\left(\sigma_{q}^{new}\right)}^{2}R_{k}}\cdot {\left(R_{k}-q^{new}\cdot (S_{k}^{-}-S_{k}^{+})\right)}^{2}〉=0[\sigma_{q}^{new}]$$

(17) $$\sum_{k=1}^{M-1}\frac{\partial l_{k}}{\partial \tau_{D}}\left[〈\frac{S_{k+1}^{-}}{l_{k}(1-l_{k})}〉-〈\frac{S_{k}^{+}}{l_{k}}〉-\frac{N}{1-l_{k}}\right]=0[\tau_{D}^{new}]$$

(18) $$\sum_{k=1}^{M}\frac{\partial u_{k}}{\partial U}\left[〈\frac{S_{k}^{-}}{u_{k}}〉-〈\frac{S_{k}^{+}}{u_{k}(1-u_{k})}〉\right]=0[U^{new}]$$

(19) $$\sum_{k=1}^{M}\frac{\partial u_{k}}{\partial \tau_{F}}\left[〈\frac{S_{k}^{-}}{u_{k}}〉-〈\frac{S_{k}^{+}}{u_{k}(1-u_{k})}〉\right]=0.[\tau_{F}^{new}].$$

Note that *u_k_* and *l_k_* depend on the new estimate *U^new^*, $\tau_{F}^{new}$, and $\tau_{D}^{new}$. The angular brackets denote average over the distribution $P(S_{1\to M}^{-},S_{1\to M}^{+}|R_{1\to M},\theta^{old})$ (see Eq. 13). This average has the following convenient property:

(20) $$\begin{array}{l} 〈g(S_{k}^{-},S_{k}^{+})〉=\sum_{S_{1\to M}^{-}}\sum_{S_{1\to M}^{+}}g(S_{k}^{-},S_{k}^{+})\cdot P(S_{1\to M}^{-},S_{1\to M}^{+}|R_{1\to M},\theta^{old})\\ =\sum_{S_{k}^{-},S_{k}^{+}}g(S_{k}^{-},S_{k}^{+})\cdot P(S_{k}^{-},S_{k}^{+}|R_{1\to M},\theta^{old}). \end{array}$$

which holds for any function, *g*. The conditions for *U^new^* and $\tau_{F}^{new}$ involve *u_k_* and its derivatives, which in turn depend on *U^new^* and $\tau_{F}^{new}$. These conditions thus have to be evaluated simultaneously.

In the presence of Gaussian baseline noise (independent of the number of released vesicles), Equation 11 becomes the following:

(21) $$P(R_{k}|S_{k}^{+},S_{k}^{-})=\int_{0}^{+\infty }\frac{dy}{\sigma_{n}\sqrt{2\pi }}P(y|S_{k}^{+},S_{k}^{-})\cdot \exp[-\frac{{\left(R_{k}-y\right)}^{2}}{2\sigma_{n}^{2}}].$$

where $\sigma_{n}^{2}$ is the estimated variance of the baseline noise (see the Preprocessing section). When the above equation is used to derive the EM re-estimation formulas, the equations for *q^new^* and $\sigma_{q}^{new}$ read as follows:

(22) $$\sum_{k=1}^{M}〈\frac{\int_{0}^{+\infty }y\cdot I(y)dy}{\int_{0}^{+\infty }I(y)dy}-q^{new}\cdot (S_{k}^{-}-S_{k}^{+})〉=0[q^{new}]$$

(23) $$\sum_{k=1}^{M}〈\frac{\int_{0}^{+\infty }{\left(y-q^{new}\cdot (S_{k}^{-}-S_{k}^{+})\right)}^{2}\cdot y^{-1}\cdot I(y)dy}{\int_{0}^{+\infty }I(y)dy}-\frac{{\left(\sigma_{q}^{new}\right)}^{2}}{q^{new}}〉=0[\sigma_{q}^{new}].$$

with *I*(*y*) given by:

(24) $$I(y)=y^{-\frac{3}{2}}\cdot \exp\{-\frac{q\cdot {\left[y-q\cdot (S_{k}^{-}-S_{k}^{+})\right]}^{2}}{2\sigma_{q}^{2}y}-\frac{{\left(R_{k}-y\right)}^{2}}{2\sigma_{n}^{2}}\}.$$

Re-estimation formulas have to be evaluated numerically. The EM algorithm was implemented in C++. For multidimensional root finding, we used the Brent-Dekker algorithm and a derivative-free version of Powell’s hybrid algorithm, as provided by the GNU Scientific Library (Galassi et al., 2009). The code is available from the authors upon request.

### Forward-backward formalism

To efficiently compute the averages over $P(S_{1\to M}^{-},S_{1\to M}^{+}|R_{1\to M},\theta^{old})$, we developed a forward-backward scheme (Rabiner, 1989). We define two forward variables as follows:

(25) $$\alpha_{k}^{-}(S)=P(S_{k}^{-}=S,R_{1\to k-1})$$

(26) $$\alpha_{k}^{+}(S)=P(S_{k}^{+}=S,R_{1\to k}),$$

where we have dropped the dependence of the parameters to simplify the notation. These variables can be evaluated recursively as follows:

(27) $$\alpha_{1}^{-}(S)=P(S_{1}^{-}=S)$$

(28) $$\alpha_{k}^{+}(S)=\overset{N}{\underset{S_{k}^{-}=0}{\sum }}\alpha_{k}^{-}(S)P(S_{k}^{+}=S|S_{k}^{-})P(R_{k}|S_{k}^{+}=S,S_{k}^{-})\;$$

(29) $$\alpha_{k+1}^{-}(S)=\sum_{S_{k}^{+}=0}^{N}\alpha_{k}^{+}(S)P(S_{k+1}^{-}=S|S_{k}^{+}).$$

Note that $\alpha_{M}^{+}(S)=P(S_{M}^{+}=S,R_{1\to M})$ and thus $\sum_{S=0}^{N}\alpha_{M}^{+}(S)=P(R_{1\to M})$. Similarly, we can define two backward variables as follows:

(30) $$\beta_{k}^{-}(S)=P(R_{k\to M}|S_{k}^{-}=S)$$

(31) $$\beta_{k}^{+}(S)=P(R_{k+1\to M}|S_{k}^{+}=S),$$

which can be evaluated recursively as follows:

(32) $$\beta_{1}^{+}(S)=1$$

(33) $$\beta_{k}^{-}(S)=\overset{N}{\underset{S_{k}^{+}=0}{\sum }}\beta_{k}^{+}(S)P(S_{k}^{+}|S_{k}^{-}=S)P(R_{k}|S_{k}^{+},S_{k}^{-}=S)\;$$

(34) $$\beta_{k-1}^{+}(S)=\sum_{S_{k}^{-}=0}^{N}\beta_{k}^{-}(S)P(S_{k}^{-}|S_{k-1}^{+}=S),$$

and $\sum_{S=0}^{N}\beta_{1}^{-}(S)P(S_{1}^{-}=S)=P(R_{1\to M})$. From this, it follows that the conditional distribution appearing in Equation 20 can be easily computed from the following:

(35) $$P\left(S_{k}^{-},S_{k}^{+}|R_{1\to M},\theta \right)=\frac{\beta_{k}^{+}(S_{k}^{+})\cdot P(R_{k}|S_{k}^{-},S_{k}^{+})\cdot P(S_{k}^{+}|S_{k}^{-})\cdot \alpha_{k}^{-}(S_{k}^{-})}{P(R_{1\to M})}.$$

### Fisher information matrix

To quantify the amount of information about a specific parameter that a stimulation protocol allows one to extract, we can compute the Fisher Information Matrix (FIM) of the generative model

(36) $$I{\left(\theta \right)}_{j,k}=E\left[\left(\frac{\partial }{\partial \theta_{j}}\log\left\{P(R_{1\to M}|t_{1\to M},\theta )\right\}\right)\times \left(\frac{\partial }{\partial \theta_{k}}\log\left\{P(R_{1\to M}|t_{1\to M},\theta )\right\}\right)|\theta \right],$$

where we have introduced explicitly the dependence on $t_{1\to M},\;E[\cdot ]$ that denotes the expectation value over the responses sequences, and *j* and *k* denote (continuous) model parameters. The diagonal elements of the inverse FIM are lower bounds on the variances of the corresponding parameters estimate. Hence, a lower bound on the relative error *ϵ* of the estimate of *θ_j_* can be written as follows:

(37) $$\epsilon (\theta_{j})\geq \frac{\sqrt{{\left[I{\left(\theta \right)}^{-1}\right]}_{jj}}}{\theta_{j}},$$

which is just a normalized version of the Cramér-Rao bound (Dayan and Abbott, 2001).

The calculation of the derivatives in Equation 36 can be performed efficiently using the forward variables $\alpha_{k}^{\pm }(S)$. We can write the following:

(38) $$\frac{\partial }{\partial \theta_{j}}\log\left[P\left(R_{1\to M}|t_{1\to M},\theta \right)\right]=\frac{1}{P\left(R_{1\to M}|t_{1\to M},\theta \right)}\cdot \sum_{S=0}^{N}\frac{\partial \alpha_{M}^{+}(S)}{\partial \theta_{j}}.$$

Since $\alpha_{k}^{+}(S)$ depends on $\alpha_{k}^{-}(S)$ and vice versa, we obtain recursive formulas for their respective derivatives, as follows:

(39) $$\frac{\partial }{\partial \theta_{j}}\alpha_{k}^{+}(S)=\sum_{S_{k}^{-}=0}^{N}\alpha_{k}^{-}(S_{k}^{-})\cdot P(S_{k}^{+}=S|S_{k}^{-})\cdot \left\{\frac{\partial P(R_{k}|S_{k}^{-},S_{k}^{+}=S)}{\partial \theta_{j}}\right\}+\sum_{S_{k}^{-}=0}^{N}\alpha_{k}^{-}(S_{k}^{-})\cdot \left\{\frac{P(S_{k}^{+}=S|S_{k}^{-})}{\partial \theta_{j}}\right\}\cdot P(R_{k}|S_{k}^{-},S_{k}^{+}=S)+\sum_{S_{k}^{-}=0}^{N}\left\{\frac{\partial \alpha_{k}^{-}(S_{k}^{-})}{\partial \theta_{j}}\right\}\cdot P(S_{k}^{+}=S|S_{k}^{-})\cdot P(R_{k}|S_{k}^{-},S_{k}^{+}=S),$$

(40) $$\begin{array}{l} \frac{\partial }{\partial \theta_{j}}\alpha_{k}^{-}(S)=\sum_{S_{k-1}^{+}=0}^{N}\alpha_{k-1}^{+}(S_{k-1}^{+})\cdot \left\{\frac{\partial P(S_{k}^{-}=S|S_{k-1}^{+})}{\partial \theta_{j}}\right\}\\ +\sum_{S_{k-1}^{+}=0}^{N}\left\{\frac{\partial \alpha_{k-1}^{+}(S_{k-1}^{+})}{\partial \theta_{j}}\right\}\cdot P(S_{k}^{-}=S|S_{k-1}^{+}). \end{array}$$

### Least-squares fitting

To fit the average model response ${\bar{R}}_{1\to M}$ to the average experimental responses ${〈R〉}_{1\to M}$, we used a standard least-squares procedure (see, e.g., Tsodyks and Markram, 1997; Markram et al., 1998), as follows:

(41) θ^LS=arg minθLS∑i=1M(〈R〉i−R¯i)2si2.

where the ${\bar{R}}_{i}$ values depends on the parameters $\theta_{LS}=\{A,U,\tau_{D},\tau_{F}\}$ (see Eqs. 5–7), and $s_{i}^{2}$ denotes the variance of the *i*th response.

### Least-squares condition number

The least-squares fitting is a mapping from the average experimental responses ${〈R〉}_{1\to M}$ to the four parameters $\theta_{LS}=\{A,U,\tau_{D},\tau_{F}\}$, as follows:

(42) $$\theta_{LS}=F({〈R〉}_{1\to M}).$$

The condition number (Trefethen and Bau, 1997) of this mapping is given by the following:

(43) $$c=\frac{∥D∥\cdot ∥{\overset{¯}{R}}_{1\to M}∥}{∥\theta_{LS}∥},$$

where ∥⋅∥ denotes the 2-norm and *D* is a 4 × *M* matrix whose elements are as follows:

(44) $$D_{ij}=\frac{\partial F_{i}}{\partial {〈R〉}_{j}}=\frac{\partial \theta_{LS}^{\left(i\right)}}{\partial {〈R〉}_{j}}.$$

The *D_ij_* can be straightforwardly evaluated numerically. The condition number measures the sensitivity of the estimates to small changes in the average responses. If c > 1, small perturbations in the average responses will tend to cause disproportionately large changes in the estimates of the parameters. In that case, the estimation procedure is ill posed.

## Results

### Generative model description of repetitive synaptic transmission: overview

We provide here a compact presentation of our method omitting unnecessary technical details. The full presentation can be found in Materials and Methods.

Synaptic transmission is, at least in the experimental conditions in which it is routinely probed, stochastic so that recorded postsynaptic responses will vary across repetitions of the same presynaptic stimulation. This variability is not just noise, but rather it carries important information about the synaptic dynamics. Accordingly, our approach seeks to select synaptic parameters so as to best describe the observed variability of the responses, instead of their averages. This is done by constructing a generative model of repetitive synaptic transmission, i.e. a parametric probability distribution function for the trains of postsynaptic responses given the times of presynaptic activation, and then determining the corresponding parameters by the maximum-likelihood principle.

**Figure 1.**
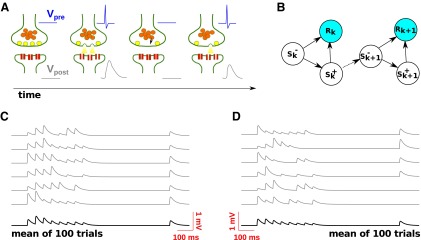
The generative model and sample synthetic traces. ***A***, Schematics of the synaptic model. Upon spike (blue), only the docked vesicles (yellow) can be released. The postsynaptic response (gray) is proportional on average to the number of vesicles released. In between spikes, vesicles dock to noncompetent release sites (black arrow) with constant probability per unit time. ***B***, Graphical model of the statistical dependencies among the number of docked vesicles before spike (*S^−^*) and after spike (*S* ^+^) and observable responses (*R*). The number of docked vesicles is not observable. ***C***, Sample synthetic traces and average trace for a facilitating connection: *N* = 10, *q* = 0.15 mV, *σ_q_* = 0.03 mV, *U* = 0.3, *τ_D_* = 195 ms, *τ_F_* = 570 ms. ***D***, Same as ***C*** for a depressing connection: *N* = 10, *q* = 0.15 mV, *σ_q_* = 0.03 mV, *U* = 0.25, *τ_D_* = 670 ms, *τ_F_* = 15 ms.

We build a stochastic generative model of synaptic transmission by following a standard procedure which consists in augmenting the quantal model with dynamic processes that modulate the total release probability. According to the quantal model, a synaptic connection is a collection of *N* independent release sites. Upon the spike, a site can either release neurotransmitter or fail to do so. The probability of such an event (i.e., the total release probability) is decomposed into the product of the probability that the site is release competent and the probability that the release actually occurs, given that the site is release competent (Quastel, 1997). Hereafter, we refer to this latter simply as the release probability. A large class of stochastic models of synaptic transmission can be formulated in this framework (indeed, all the models we are aware of), by appropriately selecting the dynamics of the release site and of the release probability (Fig. 1*A*). In the following, we chose the specific instantiations of those dynamics that correspond to the stochastic TM model (Fuhrmann et al., 2002).

In the stochastic TM model, the dynamics of the release site are described by the following simple docking process: a noncompetent site becomes competent with a constant probability per unit time 1/*τ_D_*. A competent site becomes noncompetent by releasing upon spike. The probability of release *u*(*t*) increases with each spike and decays back to its baseline level *U*, with a time constant *τ_F_* in between spikes. The dynamics of *u*(*t*) are a minimal phenomenological description of the effects of calcium influx into the synaptic terminal on the probability of release (Bertram et al., 1996; Markram et al., 1998; Dittman et al., 2000; Neher and Sakaba, 2008). The postsynaptic response to a single vesicle (quantal response) is variable with mean *q* (quantal size) and variance $\sigma_{q}^{2}$ (quantal noise). The postsynaptic response to multiple vesicles is simply the sum of the single quantal responses.

This model has two dynamic variables—the number of release-competent sites *S*(*t*) and the release probability *u*(*t*)—and six parameters—the number of release sites *N*, the quantal size *q*, the quantal noise $\sigma_{q}^{2}$, the initial release probability *U*, and the time constants for docking and facilitation, *τ_D_* and *τ_F_*, respectively. It contains three sources of variability since both release and docking are stochastic processes, and the same number of released vesicles can result in different postsynaptic responses due to quantal noise. These sources of variability are all well documented experimentally. In Figure 1, we show sample synthetic traces generated by the model, together with the trial-averaged trace, for a facilitating connection (Fig. 1*C*) and a depressing connection (Fig. 1*D*).

Having an explicit description for the sources of stochasticity, one can compute the probability that a given train of postsynaptic responses, $R_{1\to M}\equiv \{R(t_{1}),R(t_{2}),\cdots \;,R(t_{M})\}$, is observed in correspondence with presynaptic spikes occurring at times $t_{1\to M}\equiv \{t_{1},t_{2},\cdots \;,t_{M}\}$. This probability as a function of the model parameters, which we collectively denote θ, is by definition the likelihood function, i.e.,

(45) $$L(\theta |R_{1\to M})\equiv P(R_{1\to M}|\theta ).$$

The maximum-likelihood principle prescribes taking as an estimate of the parameters the values ${\hat{\theta }}_{\text{ML}}$, which maximize $L(\theta |R_{1\to M})$. Although the likelihood function can be efficiently evaluated numerically (as we show in Materials and Methods), its direct maximization turns out to be impractical because the different responses are correlated, (i.e., $P(R_{1\to M}|\theta )\neq \prod_{k=1}^{M}P(R_{k}|\theta )$). The origin of these correlations is easy to understand. Let us denote $S_{1\to M}^{-}\equiv \{S(t_{1}^{-}),S(t_{2}^{-}),\cdots \;,S(t_{M}^{-})\}$ and $S_{1\to M}^{+}\equiv \{S(t_{1}^{+}),S(t_{2}^{+}),\cdots \;,S(t_{M}^{+})\}$ the number of release-competent sites immediately before and after the corresponding spikes, respectively. The probability of observing a given response, *R_k_*, depends on the number of vesicles released upon the *k*th spike; that is, it depends on both $S_{k}^{+}$ and $S_{k}^{-}$ (in fact, it depends on their difference). Similarly, the probability of the next response being $R_{k+1}$ depends on both $S_{k+1}^{+}$ and $S_{k+1}^{-}$. However, the probability of having a given number of release-competent sites $S_{k+1}^{-}$ immediately before the (*k* + 1)-th spike depends on the number of release-competent sites $S_{k}^{+}$ immediately after the *k*th spike (Fig. 1*B*). These probabilistic dependencies, which are graphically illustrated in Figure 1*B*, are the source of the correlations between the different synaptic responses. Note that, if properly dealt with, these correlations are a source of information about the underlying synaptic dynamics rather than a hindrance.

It should be clear from the above that the responses *R*_1→_*_k_* and *R_k_*_+1→_*_M_* are independent, conditionally on the knowledge of $S_{k}^{+}$ (Fig. 1*B*). Thus, the joint probability of the observed responses and the underlying sequence of the numbers of release-competent sites responsible for their generation can be conveniently factorized in the following way:

(46) $$P(R_{1\to M},S_{1\to M}^{-},S_{1\to M}^{+}|\theta )=P(S_{1}^{-}|\theta )\prod_{k=1}^{M}P(S_{k}^{+}|S_{k}^{-},\theta )P(R_{k}|S_{k}^{+},S_{k}^{-},\theta )\prod_{k=1}^{M-1}P(S_{k+1}^{-}|S_{k}^{+},\theta ).$$

The conditional probabilities appearing in the above equation have the following straightforward interpretation: $P(S_{1}^{-}|\theta )$ is the initial distribution of the number of release-competent sites; $P(S_{k}^{+}|S_{k}^{-},\theta )$ is the probability that the number of release-competent sites changes from $S_{k}^{-}$ to $S_{k}^{+}$ upon spike; $P(R_{k}|S_{k}^{+},S_{k}^{-},\theta )$ is the probability of observing a postsynaptic response *R_k_* when the number of release-competent sites changes from $S_{k}^{-}$ to $S_{k}^{+}$; and $P(S_{k+1}^{-}|S_{k}^{+},\theta )$ is the probability that the number of release-competent sites changes from $S_{k}^{+}$ to $S_{k+1}^{-}$ during the time interval $t_{k+1}-t_{k}$ in the absence of spikes. These conditional probabilities are easily computed from the model.

Unlike synaptic responses, however, the number of release-competent sites is not directly observable (i.e., is a hidden variable). A very powerful algorithm, the EM algorithm (Dempster et al., 1977; Dayan and Abbott, 2001), exists that allows a maximum-likelihood estimation in the presence of hidden variables, as it is our case. In Materials and Methods, we show how all the quantities needed to carry out EM can be efficiently computed, and we obtain explicit re-estimation formulas for the parameters.

## Application to experimental data

### Response variability

**Figure 2.**
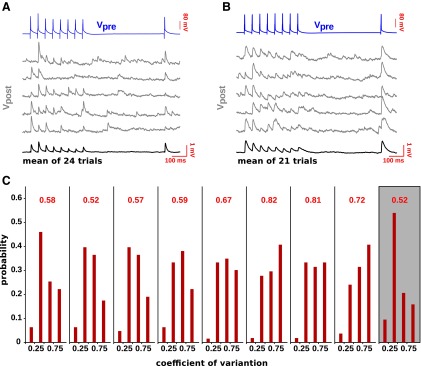
Stimulation protocol and variability of synaptic responses (experimental data). ***A***, Five sample single-trial trains of experimentally measured postsynaptic responses (gray traces) illustrate the large trial-to-trial variability. The trial-averaged trace (black trace) reveals facilitating transmission (compare first and second response). Stimulation consists of a regular train of eight spikes followed by a recovery spike (top blue trace). ***B***, Same as in ***A*** for a depressing connection (compare first and second responses). ***C***, Histograms of the CVs of the synaptic responses in the train and upon the recovery spike (gray-shaded panel) across the dataset. The red numbers denote the respective average CVs. The size of the bins is 0.25, apart from the last one, which includes all CVs >0.75.

We began by analyzing response variability, which was not done in the study by Wang et al. (2006), and found that the synaptic responses exhibited strong variability. For the purpose of illustration, we show in Figure 2 sample voltage traces for one facilitating connection (Fig. 2*A*) and one depressing connection (Fig. 2*B*), together with the corresponding trial-averaged traces. In both cases, the large variability across the different repetitions is immediately evident. The variability is, indeed, so strong that the facilitating/depressing nature of the transmission is largely concealed in the single traces, while it becomes readily apparent in the trial-averaged traces.

To quantify variability, for each connection we extracted from the corresponding single-trial voltage traces the peak postsynaptic response corresponding to each presynaptic spike, as explained in Materials and Methods. For each connection, we then computed the coefficient of variation (CV) of each response, which was defined as the ratio between the SD of the response across the different trials and its average. The results of this analysis are shown in Figure 2*C*, where we report the histograms of the CV across the population of synaptic connections, separately for each response. The average of the initial response ranged between 0.11 and 3.43 mV (0.67 ± 0.58 mV; *n* = 69), while the associated CV ranged between 0.21 and 1.58 (0.59 ± 0.27; *n* = 69). These values are fully consistent with those from previous studies (Markram et al., 1997; Brémaud et al., 2007; Loebel et al., 2009). We took this as an indication of the reliability of the procedure we used for isolating synaptic responses within the single-trial voltage traces.

Synaptic unreliability remained high all along the stimulation, and it even increased for late responses, as can be seen in Figure 2*C*. This is a consequence of the increasing probability of failure due to vesicle depletion (Loebel et al., 2009). The population-averaged CV was smallest for the second and the recovery response. This is a consequence of the increasing probability of release occurring at facilitating synapses, while release-competent sites are still abundant (i.e., before depression builds up). The second and the recovery responses were, in fact, the most facilitated responses on average. Note that such high levels of variability are the rule, rather than the exception, for central chemical synapses (Markram et al., 1997; Brémaud et al., 2007; Loebel et al., 2009).

Two points are worth stressing. As we just discussed, changes in the level of variability of the responses carry information about the underlying synaptic dynamics. This information, however, is destroyed by the trial-averaging procedure needed to perform least-squares fitting. Second, the precision of the least-squares estimates for the parameters is fundamentally limited by the accuracy of the (experimental) estimates of the average responses. A straightforward application of the central limit theorem shows that, for a true CV of 0.3, an estimate of the average response within 10% relative precision would require about 80 repetitions, while an estimate within 5% relative precision would require >300 repetitions. Given the CVs estimated from the data, these figures should be considered as the minimal number of repetitions needed to estimate the average response with reasonable accuracy. Unfortunately, least-squares estimates can be grossly imprecise even when the empirical averages are determined with high accuracy, as we show in the Maximum-likelihood versus least-squares estimation section.

### Maximum-likelihood estimation of the synaptic parameters

The synaptic parameters $\theta =\left\{N,q,\sigma_{q},U,\tau_{D},\tau_{F}\right\}$ for a given connection were estimated as follows. For a fixed value of *N* (note that *N* takes on only integer values, while the other parameters are continuous), the maximum-likelihood estimate of the remaining parameters is obtained by using the EM algorithm (see Materials and Methods). We varied *N* between 1 and 100, and the value of *N* and of the corresponding continuous parameters for which the likelihood was maximal was then selected as the final estimate.

**Figure 3.**
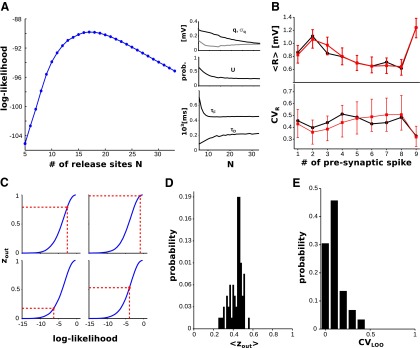
Maximum-likelihood estimation of the synaptic parameters from experimental data. ***A***, Log-likelihood (left) and associated model parameters (right) as a function of *N* for a sample connection. The maximum is attained at *N* = 17 with *q* = 0.18 mV, *σ_q_* = 0.06 mV, *U* = 0.27, *τ_D_* = 202 ms, *τ_F_* = 449 ms. ***B***, Top, Average experimental responses (black line) vs average model responses (red line) for the same connection as in ***A***. Bottom, Same as in the top panel for the coefficients of variation. Error bars indicate the 95% confidence interval of the model prediction. ***C***, Cumulative distribution function of the log-likelihood for four instances of the leave-one-out procedure (blue curves). The red dots indicate the log-likelihood of the left-out trials. Same connection as in ***A***. ***D***, Distribution over the dataset of the average *z*_out_. ***E***, Distribution over the dataset of the average coefficients of variation of the estimates obtained in the leave-one-out procedure.

The procedure is illustrated in Figure 3*A* for a sample connection. In Figure 3*A*, left, we plot the log-likelihood as a function of *N*, while, in Figure 3*A*, right panels, we plot the values of the parameters that maximize the log-likelihood for the corresponding *N*. The log-likelihood exhibits a clear maximum at *N* = 17. The values of the remaining parameters can be read from the corresponding curves on the right. They are as follows: *q* = 0.18 mV, *σ_q_* = 0.06 mV, *U* = 0.27, *τ_D_* = 202 ms, and *τ_F_* = 449 ms. Using these parameters, we generated 500 synthetic experiments in which the model was probed with the same stimulation protocol, and for the same number of trials (28), as in the real experiment. We then computed the average responses and the associated CVs for each experiment, and from these the corresponding grand averages together with 95% confidence intervals. The results are shown in Figure 3*B*. In the top panel of Figure 3*B*, we report the experimental (black curve) and the model average responses (red curve; error bars represent 95% confidence intervals). In the bottom panel of Figure 3*B*, we report the experimental (black curve) and the synthetic CVs (red curve; error bars represent 95% confidence interval). The model parameters were not selected to reproduce the average responses or the CVs, but rather to maximize the probability of the occurrence of the actual trains of responses observed in the experiment. Nevertheless, as can be seen, the experimental data are well reproduced by the model.

We estimated the synaptic parameters for all the connections in our dataset. In 6 of 69 cases, the estimation procedure returned values for one or more parameters that were judged to be problematic. In four cases, the estimation procedure returned values for one or both the time constants (i.e., *τ_D_* and *τ_F_*) that were several orders of magnitude larger than the longest timescale at which the synaptic connections were probed. In the two remaining cases, there was no maximum in the range of *N* values probed. These connections were excluded from further analysis.

We checked next whether the model was overfitting the data by using a standard leave-one-out cross-validation procedure (Stone, 1974). We estimated the parameters as described above while leaving out one trial every time. With the parameters obtained, we computed the probability that the model would generate a set of responses with a log-likelihood equal or smaller than the log-likelihood of the set of responses that was left out. Hereafter, we denote this probability by *z*_out_. This procedure is illustrated in Figure 3*C* for the same connection as in Figure 3*A*. In each subpanel, we show (1) the cumulative distribution of the log-likelihood for a set of responses generated from the model, where the parameters are estimated by leaving out one trial (blue curve); (2) the value of the log-likelihood of the set of responses left out during the estimation procedure (on the *x*-axis); and (3) the corresponding value of *z*_out_ (on the *y*-axis). For sets of responses generated by the model, one expects *z*_out_ to be uniformly distributed between 0 and 1. The distribution of the average (over all trials) *z*_out_ value across the dataset is shown in Figure 3*D*. As can be seen, most of the values are between 0.4 and 0.5. For only 5 of 63 connections, the distribution of *z*_out_ values obtained from the leave-one-out procedure was statistically different from the uniform distribution (Kolmogorov–Smirnov test, *p* = 0.01). For three of these connections, <25 trials were available. No connection with ≥30 trials (*n* = 10) showed a distribution of *z*_out_ values that was statistically different from the uniform distribution.

The leave-one-out procedure also allowed us to evaluate the stability of the estimation procedure, which we quantified by computing the average coefficient of variation of the estimates. The distribution obtained is reported in Figure 3*E*.

We concluded that, for the large majority of the connections (58 of 63), there were no indications of overfitting, while for the remaining connections it was likely that we simply lacked statistical power to assess overfitting. Also our estimation procedure exhibited an accuracy and stability that tended to be better than the least-squares fitting procedure (see the Maximum-likelihood versus least-squares estimation section), while allowing us to estimate two additional parameters.

### Quantifying the uncertainty of the parameter estimates

**Figure 4.**
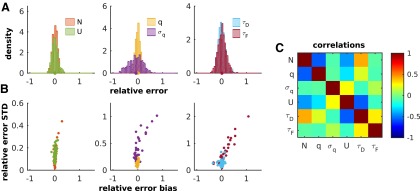
Estimating uncertainty and correlations between parameter estimates with parametric bootstrap. ***A***, Distributions of the relative errors obtained by re-estimating the parameters of the sample connection in Fig. 3*A* from synthetically generated responses. The dots on the *x*-axes indicate the corresponding averages. ***B***, Standard error vs bias of the relative error for all the connections. ***C***, Pearson correlation coefficients between all pairs of parameter estimates averaged over all the connections.

We used a standard re-sampling procedure (i.e., parametric bootstrap; Efron and Tibshirani, 1994) to quantify the uncertainty of the parameter estimates. For each set of parameters obtained from a synaptic connection in our dataset, we generated 500 synthetic experiments with the same settings as in the original experiment (i.e., same stimulation protocol, number of repetitions, and baseline noise). We then re-estimated the parameters for each synthetic experiment and computed their relative errors with respect to the original set of parameters. We show in Figure 4*A* the resulting distributions of relative errors for the same connection shown in Figure 3*A*. All the distributions peaked at ∼0, with averages very close to 0 and relative SDs <0.3. This shows that, at least for this connection, our method returns unbiased and accurate estimates of the parameters.

We see in Figure 4*B*, where we plot the SD versus the bias of the relative error for each parameter and each connection, that this is, in fact, the case for the majority of connections in our dataset. For some connections, however, the estimates of *σ_q_* and/or *τ_F_* are quite inaccurate. Large relative errors in the estimates of *σ_q_* are due to the connections for which the baseline noise is comparable or larger than the quantal noise. Large relative errors in the estimates of *τ_F_* are due to connections for which the (experimentally) chosen interstimulus interval was ill suited to resolve the facilitation time course. For instance, in depression-dominated connections (i.e., *τ_F_* < *τ_D_*) the facilitation time constant cannot be reliably estimated if the interstimulus interval is much longer than *τ_F_*. The high uncertainty in the estimates of *σ_q_* and *τ_F_* (for some connections) is responsible for the positive biases observed in Figure 4*B*. This is because relative errors cannot be smaller than −1, given that both parameters have to be positive, while they can be quite large and positive (i.e., ≫1). It is important to stress that the large variability and the accompanying bias in the estimates of *σ_q_* and *τ_F_* are not due to the method itself but to the small amount of information conveyed by the data (experimental or synthetic). This can be remedied, especially for *τ_F_*, by choosing more informative stimulation protocols (see the Estimating synaptic parameters from a single spike train section).

Using the data generated in the synthetic experiments, we also computed, for each connection, the (Pearson) correlation coefficients between all pairs of parameter estimates. The correlation coefficients were then Fisher transformed (Efron and Tibshirani, 1994), averaged across connections, and Fisher transformed back. The result is shown in Figure 4*C*. Fluctuations in the parameter estimates are all significantly correlated. This is to be expected. An estimate that deviates from its true value necessarily induces compensatory adjustments in one or more of the other parameters. For instance, the overestimation of *N* must be compensated by the underestimation of *q* (or *U* or both) in order to reproduce the empirically observed range of the responses. In fact, correlations between *N* and *q*, *N* and *U*, and *U* and *q* were all negative and quite substantial (*R* = −0.56, *R* = −0.44, and *R* = −0.32, respectively). Similarly, the underestimation of *N* must be compensated by the underestimation of *τ_D_* (i.e., faster recovery from depression) in order to maintain the empirically observed range of responses throughout the train. Accordingly, *N* and *τ_D_* are positively correlated (*R* = 0.40).

### Maximum-likelihood estimation versus least-squares fitting

**Figure 5.**
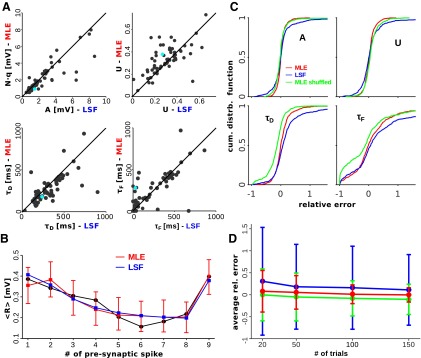
Maximum-likelihood estimation (MLE) vs least-squares fitting (LSF). ***A***, Estimates obtained with MLE vs those obtained with LSF for all the connections in the dataset. ***B***, Average experimental responses (black line) for the connection corresponding to the cyan dot in ***A*** together with the result of LSF (blue line) and the MLE prediction with 95% confidence interval (red line + bars). MLE estimate: *A* = 0.91 mV, *U* = 0.38, *τ_D_* = 179 ms, *τ_F_* = 279 ms; LSF estimate: *A* = 1.48 mV, *U* = 0.27, *τ_D_* = 236 ms, *τ_F_* = 28 ms. ***C***, Cumulative distribution functions of the relative errors of MLE (red), MLE with shuffled responses (green) and LSF (blue) estimates from synthetic experiments. ***D***, Average and SD (bars) of relative errors for MLE (red), MLE with shuffled responses (green), and LSF (blue) estimates as a function of the number of trials (synthetic experiments).

Standard least-squares fitting allows one to estimate the initial release probability *U*, the two time constants *τ_D_* and *τ_F_*, and the product *N* ⋅ *q*, which we denote by *A* (for details, see Materials and Methods). We thus compared the estimates obtained with our method with the ones obtained by least-squares fitting. Note that the two methods would return asymptotically the same estimates for these parameters. In Figure 5*A*, we plot the estimates obtained with our method versus the estimates obtained with the least-squares fitting procedure. As can be seen, the two methods tend to return correlated estimates. Nevertheless, for more than half of the connections, the relative difference between the estimates with the two methods was >30% for at least one of the parameters. In several cases, the two estimates were dramatically different. One such case is illustrated in Figure 5*B*. According to maximum-likelihood estimation, this connection was facilitating (*τ_F_* = 279 ms, *τ_D_* = 179 ms, *τ_F_*/*τ_D_* = 1.56), while according to least-squares fitting the same connection was depressing (*τ_F_* = 28 ms, *τ_D_* = 236 ms, *τ_F_*/*τ_D_* = 0.12). Note that the estimates of *τ_F_* differed by one order of magnitude. The large differences between the estimates with the two methods thus suggest that, for most of the connections, the number of repetitions is too small to reach the asymptotic regime.

Our method makes use of more information (i.e., correlations and variability) to estimate the parameters. One would accordingly expect more accurate estimates compared with least-squares fitting, and the often quite large discrepancies would thus result from the different accuracies of the two methods. To test this hypothesis, we generated 500 synthetic connections by randomly and independently selecting their parameters from the corresponding experimental distributions (as estimated with our method; see Population analysis section). The synthetic connections were probed with a regular train of eight spikes at 20 Hz, followed by a recovery spike 550 ms after the end of the train. We collected the responses over 20 trials for each connection, and re-estimated its synaptic parameters. To investigate the impact of the correlations on the accuracy of the estimates, we also applied our method to shuffled response trains. These were obtained by randomly reassigning responses to trains while preserving their position within the train (e.g., the second response in the first train becomes the second response in the fifth train). The distributions of the relative errors, with respect to the true parameters, obtained with our method, with shuffled trains and least-squares fitting, are shown in Figure 5*C*. As expected, the estimates obtained by least-squares fitting as well as those obtained from the shuffled trains were more inaccurate than those obtained with our method. Note that the shuffling procedure introduces a systematic error in both the estimate of *τ_D_* and the estimate of *τ_F_*. This is because consecutive responses are negatively correlated when release can occur from a finite number of sites. This correlation decays exponentially over a timescale of the order of the docking time. The shuffling procedure tends to reduce the correlation between consecutive responses, which results in the systematic underestimation of *τ_D_*. This, in turn, leads to the underestimation of *τ_F_*, as the two estimates tend to be positively correlated (Fig. 4*C*).

We next investigated how the accuracy of the estimates increased with the number of trials. We repeated the analysis described above when collecting responses over 50, 100, and 150 trials. We then computed the mean and the SD of the corresponding distributions of the relative errors (Fig. 5*C*). The results are plotted in Figure 5*D* (our method, red curve; our method with shuffled trains, green curve; least-squares fitting, blue curve). For the estimates obtained with our method, the mean of the relative errors, which is already very small for 20 trials, quickly converges to 0. Likewise, the accuracy of the estimates, as measured by the SD of the relative errors (Fig. 5*D*, bars), steadily increases with the number of trials. The estimates obtained from shuffled trains and those obtained by least-squares fitting exhibited the same overall trends. Their accuracy, however, improved in a much slower way with an increasing number of trials. Note that, even for 150 trials, the accuracy was significantly smaller than the accuracy of the estimates obtained with our method.

**Figure 6.**
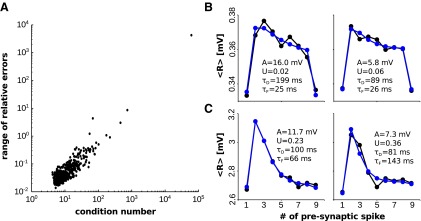
The condition number and the accuracy of the least-squares estimates. ***A***, The range of relative errors vs the condition number of the estimates obtained by least-squares fitting (synthetic experiments). ***B***, Sample synthetic average responses (black) together with average model responses (blue) resulting from the least-squares fitting. The parameters reported in the panels are the least-squares estimates. True parameters were as follows: *A* = 4.8 mV, *U* = 0.07, *τ_D_* = 95 ms, *τ_F_* = 28 ms. ***C***, Same as in ***B*** but with true parameters: *A* = 8.1 mV, *U* = 0.33, *τ_D_* = 81 ms, *τ_F_* = 100 ms.

This is particularly evident, and puzzling, for the estimates obtained by least-squares fitting. The slow increase in the accuracy of the estimates with the number of trials could be a consequence of the fact that the least-squares procedure is more prone than the maximum-likelihood procedure to get stuck in local minima (as suggested by Costa et al., 2013). Alternatively, it could result from the fact that the least-squares procedure is ill posed. To understand which of these two possibilities was the more likely explanation of the observed phenomenology, we computed, for each connection in our synthetic sample (see above), the condition number (for details, see Materials and Methods) and estimated the range of the relative errors obtained by the least-squares procedure when adding a small amount of noise to the true average responses. Concretely, for each connection we added Gaussian noise with a relative SD of 0.01 independently to each true average response in the train, and then re-estimated the parameters. We repeated this procedure 15 times, and took the largest difference between relative errors (of the same parameter) as an estimate of the corresponding range. In Figure 6*A*, we plot the range of relative errors versus the condition number. As can be seen, for a fraction of the connections the condition number, and correspondingly the range of relative errors, is quite large. For these connections, we verified that there was no global minimum in the neighborhood of the true parameters, contrary to what one would expect for a well posed least-squares procedure. Thus, large relative errors were not a result of the minimization routine getting stuck in local minima. For the purpose of illustration, we report in Figure 6, *B* and *C*, the samples least-squares fit to two connections for which the condition number was >100. As can be seen, the estimated parameters fluctuate substantially across the different noise realizations.

Although we cannot rule out definitively the possibility that the least-squares procedure returning local minima contributes to the poor increase in the accuracy of the estimates with the number of trials, we believe that that is largely a result of the fact that, for some set of parameters, the least-squares fitting is very sensitive to the specific realizations of the synaptic responses. This could happen, as we have just shown, even for experimentally unrealistic large number of trials; to achieve a 1% relative error on the average, one would need a number of trials on the order of 10^4^.

### Population analysis

**Figure 7.**
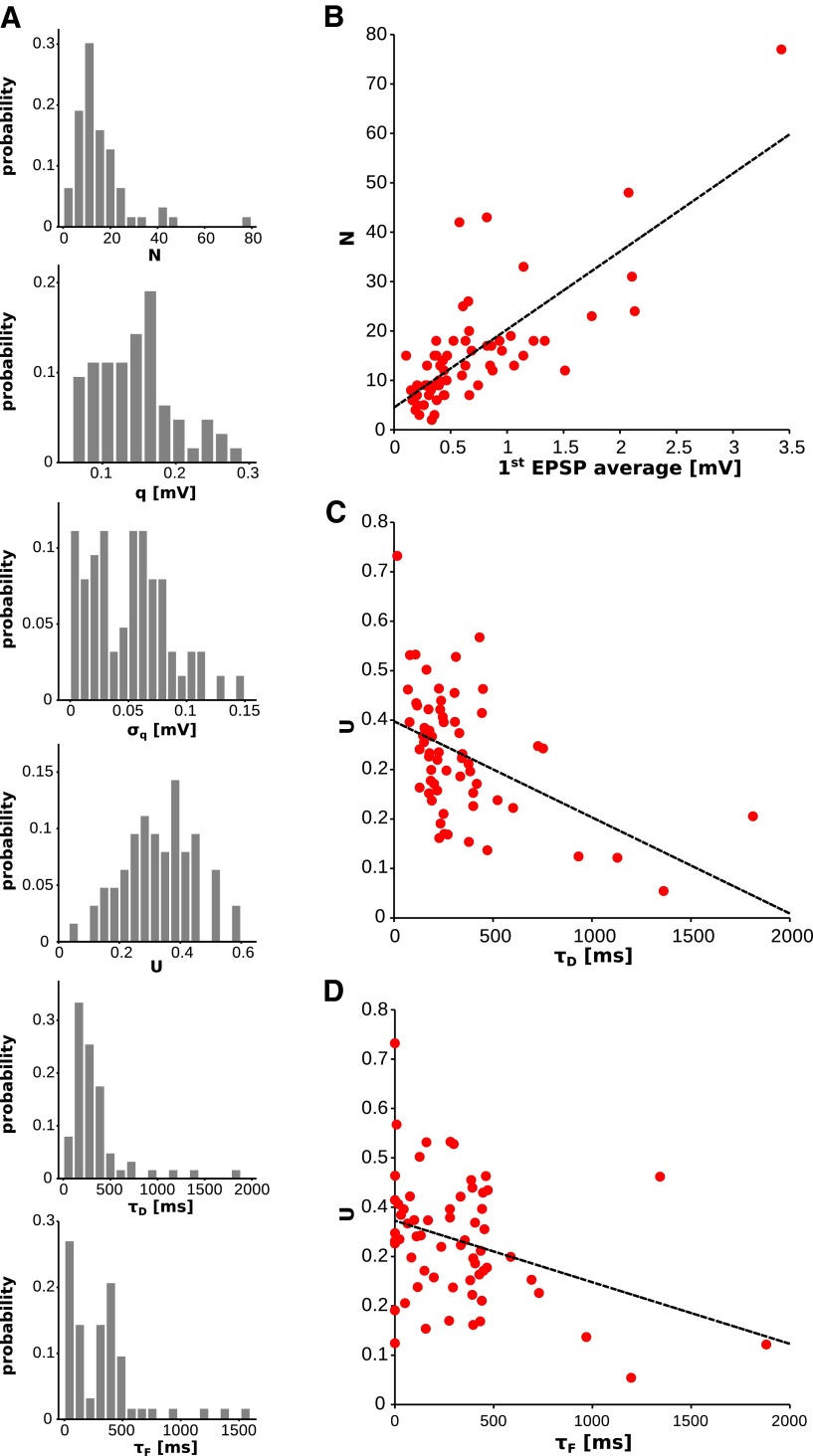
Distributions and correlations of the parameters (experimental data). ***A***, Distributions over the dataset of the different synaptic parameters. ***B***, Number of release sites *N* vs average first response in the train. Dashed line, Linear regression (*R* = 0.76, *p* < 10^−13^). ***C***, Initial release probability *U* vs time constant of the docking process *τ_D_*. Dashed line, Linear regression (*R* = −0.48, *p* < 10^−4^). ***D***, Initial release probability *U* vs time constant of facilitation *τ_F_*. Dashed line, Linear regression (*R* = −0.34, *p* < 10^−3^).

The average values and ranges of the six synaptic parameters ($N,q,\sigma_{q},U,\tau_{D},\tau_{F}$) obtained with our method are reported in Table 1, along with the estimates obtained with other methods in similar preparations and conditions (Larkman et al., 1991, 1997; Markram et al., 1998; Silver et al., 2003; Koester and Johnston, 2005; Wang et al., 2006; Brémaud et al., 2007; Loebel et al., 2009; Hardingham et al., 2010). The corresponding distributions are shown in Figure 7*A*. It is important to stress that our method returned quantal parameters in excellent agreement with published estimates, although the number of observed responses available for each connection was several times smaller than the number of observed responses typically required for current state-of-the-art methods (Silver, 2003).

**Table 1.** Parameter estimates obtained with our method (in bold type) compared with published estimates obtained in similar experimental preparations

**Parameter**	**Average ± SD**	**Range**	**Preparation**	**Temperature (°C)**	**Method**	**Reference**
	15±12	**2–77**	**Ferret mPFC, L5 to L5**	32–34	**MLE**	
	8.1 ± 5.3	3–18	Rat HC, CA1	34	H, QMLE	Larkman et al. (1997)
	5.1 ± 2.7	2.3–11.1	Rat SC, L4 spiny stellar to L2/3 pyramidal	35–37	MV	Silver et al. (2003)
*N*	7.48 ± 7.11	2–22	Rat VC and SC, various layers	35–36	MV, FA	Brémaud et al. (2007)
	12.21 ± 7.9	2–32	Cat VC, various layers	35–36	MV, FA	Brémaud et al. (2007)
	53.3 ± 42	7–170	Rat SC, L5 to L5	35	LS	Loebel et al. (2009)
	3.4 ± 2.2	1–7	Rat VC, L5 to L5	23–26/36	MV, H	Hardingham et al. (2010)
	0.15±0.06	**0.06–0.32**	**Ferret mPFC, L5 to L5**	32–34	**MLE**	
	0.131 ± 0.145	0.084–0.197	Rat HC, CA1	34	H, QMLE	Larkman et al. (1991)
	0.196 ± 0.062	0.066–0.275	Rat HC, CA1	34	H, QMLE	Larkman et al. (1997)
*q* [mV]	0.15 ± 0.09		Rat SC, L4 spiny stellar to L2/3 pyramidal	35–37	MV	Silver et al. (2003)
	0.37 ± 0.17	0.15–1	Rat VC and SC, various layers	35–36	MV, FA	Brémaud et al. (2007)
	0.22 ± 0.19	0.07–1	Cat VC, various layers	35–36	MV, FA	Brémaud et al. (2007)
	0.13 ± 0.04	0.06–0.25	Rat SC, L5 to L5	35	LS	Loebel et al. (2009)
	0.211 ± 0.065	0.106–0.302	Rat VC, L5 to L5	23–26/36	MV, H	Hardingham et al. (2010)
	0.05±0.05	**0.00–0.31**	**Ferret mPFC, L5 to L5**	32–34	**MLE**	
*σ_q_* [mV]	0.052 ± 0.085	0.030–0.10	Rat HC, CA1	34	H, QMLE	Larkman et al. (1991)
	0.065 ± 0.15		Rat SC, L4 spiny stellar to L2/3 pyramidal	35–37	MV	Silver et al. (2003)
	0.33±0.13	**0.05–0.73**	**Ferret mPFC, L5 to L5**	32–34	**MLE**	
	0.53 ± 0.17	0.14–0.81	Rat HC, CA1	34	H, QMLE	Larkman et al. (1997)
	$0.59\pm 0.16^{†}$		Rat SC, L5 to L5	32–34	LS	Markram et al. (1998)
	$0.05\pm 0.04^{‡}$		Rat SC, L5 to L5	32–34	LS	Markram et al. (1998)
	0.79 ± 0.12		Rat SC, L4 spiny stellar to L2/3 pyramidal	35–37	MV	Silver et al. (2003)
*U*	0.46 ± 0.26*	0.15–0.95	Rat SC, L2/3 to L2/3	35	OQA	Koester and Johnston (2005)
	0.27 ± 0.15	0.03–0.6	Ferret mPFC, L5 to L5	32–34	LS	Wang et al. (2006)
	0.63 ± 0.6		Rat VC and SC, various layers	35–36	MV, FA	Brémaud et al. (2007)
	0.69 ± 0.18		Cat VC, various layers	35–36	MV, FA	Brémaud et al. (2007)
	0.46 ± 0.1	0.25–0.65	Rat SC, L5 to L5	35	LS	Loebel et al. (2009)
	0.46 ± 0.21	0.15–0.75	Rat VC, L5 to L5	23–26/36	MV, H	Hardingham et al. (2010)
	0.53 ± 0.05		Rat VC, L5 to L5	32–34	Bayesian	Costa et al. (2013)
	335±306	**16–1800**	**Ferret mPFC, L5 to L5**	32–34	**MLE**	
	$813\pm 240^{†}$		Rat SC, L5 to L5	32–34	LS	Markram et al. (1998)
*τ_D_* [ms]	399 ± 295‡		Rat SC, L5 to L5	32–34	LS	Markram et al. (1998)
	396 ± 163	0–1600	Ferret mPFC, L5 to L5	32–34	LS	Wang et al. (2006)
	525 ± 134	380–900	Rat SC, L5 to L5	35	LS	Loebel et al. (2009)
	321±340	**0–1900**	**Ferret mPFC, L5 to L5**	32–34	**MLE**	
*τ_F_* [ms]	1797 ± 1247‡		Rat SC, L5 to L5	32–34	LS	Markram et al. (1998)
	292 ± 240	0–1600	Ferret mPFC, L5 to L5	32–34	LS	Wang et al. (2006)

The last three columns display the temperature at which recordings were taken, the estimation methods, and the reference to the corresponding study, respectively. All synaptic connection are pyramidal to pyramidal, unless stated otherwise. HC, Hippocampus; VC, visual cortex; SC, somato-sensory cortex; L, layer; MV, mean-variance analysis; QMLE, quantal maximum likelihood; LS, least-squares fit; H, histogram; FA, failure analysis; OQA, optical quantal analysis.

*Release probability per connection; †dominantly depressing connections; ‡dominantly facilitating connections.

The average first responses (i.e., the synaptic efficacies as routinely estimated) varied over a 32-fold range (from 0.11 to 3.43 mV) across connections. Their distribution was very well fitted by a log-normal distribution (one-sample Kolmogorov–Smirnov test, *p* ≈ 0.74; log location, −0.65; log scale, 0.73), consistent with what was reported for other cortical regions (Song et al., 2005; Loewenstein et al., 2011; Buzsáki and Mizuseki, 2014). To investigate the origin of such a large range, we searched for correlations between the synaptic efficacy and the quantal parameters *N*, *q*, and *U*. We found a very strong correlation with the number of release sites *N* (*R* = 0.76, *p* < 10^−13^; Fig. 7*B*), and a weaker but significant correlation with the quantal amplitude *q* (*R* = 0.25, *p* < 0.05). We found no correlation with the initial release probability *U*. Also, we did not find significant correlations among these three parameters. We concluded that synaptic efficacy is primarily determined by the number of release sites, which is consistent with previous reports (Loebel et al., 2009; Chabrol et al., 2015). Correlation between synaptic efficacy and quantal amplitude has also been reported previously (Hardingham et al., 2010).

Next, we searched for correlations between synaptic parameters. The initial release probability *U* was negatively correlated with both the time constant of the docking process *τ_D_* (*R* = −0.48, *p* < 10^−4^; Fig. 7*C*) and the time constant for facilitation *τ_F_* (*R* = −0.34, *p* < 10^−3^; Fig. 7*D*). It has been shown that the total number of vesicles released during a train of action potentials increases with the initial release probability (Dobrunz and Stevens, 1997). This suggests that the larger the initial release probability, the faster the docking process, which is consistent with the negative correlation between *U* and *τ_D_* that we found. Similarly, large paired-pulse ratios (i.e., strong facilitation) are typically observed in synaptic connections with a low initial release probability (Magleby, 1979; Dobrunz and Stevens, 1997; Oleskevich et al., 2000). Again, this is consistent with the negative correlation between *U* and *τ_F_* that we found. The same trends were also found with the estimates obtained with least-squares fitting. However, the correlation coefficients were weaker, and less statistically significant (*R* = −0.34, *p* = 0.007 for the correlation between *U* and *τ_D_*; *R* = −0.24, *p* = 0.06 for the correlation between *U* and *τ_F_*). We did not find other pairs of synaptic parameters exhibiting statistically significant correlation.

### Estimating synaptic parameters from a single spike train

**Figure 8.**
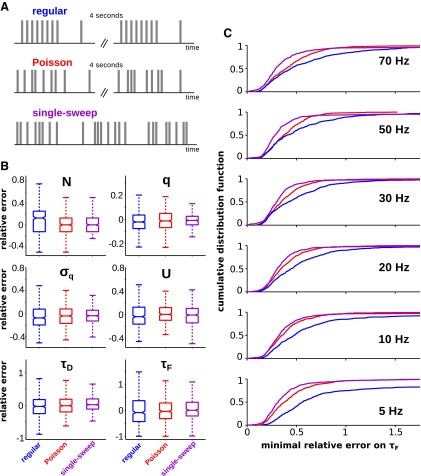
Comparison of different stimulation protocols (synthetic experiments). ***A***, Schematics of the protocols. ***B***, Box plots of the distributions of the relative errors of the estimates for all the parameters for sample synthetic connection. Stimulation frequency was 5 Hz. ***C***, Cumulative distribution function of the minimal relative errors on the estimate of *τ_F_* at varying stimulation frequencies for the regular (blue lines), the Poisson (red lines) and the single-sweep (magenta lines) protocols. Cumulative distribution functions at the same stimulation frequency are all statistically different (Kolmogorov–Smirnov test, *p* < 0.01). This is true for all stimulation frequencies.

In this section, we demonstrate one of the main advantages of our method over existing state-of-the-art methods. The least-squares fitting procedure requires the stimulation to be broken into separate trials and these trials to be repeated for putatively identical initial conditions, which is typically obtained by introducing quite long intervals (4–10 s) between consecutive trials. The method we have developed is free from both of these constraints. To illustrate the advantages this fact entails, we generated a synthetic connection by setting the parameters to the corresponding experimental population-averaged values, apart from *N*, which we set to the mode of the experimental distribution (i.e., *N* = 8), and probed it with three different stimulation protocols. The regular protocol is the same as the experimental protocol used to collect the data analyzed in this study (Fig. 2). The Poisson protocol is obtained from the regular protocol, substituting in each trial the regular train with a Poisson train with the same average interspike interval. In other words, in the Poisson protocol the spike train is different on each trial, thus removing the constraint of the repetition of the same stimulation across trials. In both the regular and Poisson protocol consecutive trials are separated by a 4 s interval. The single-sweep protocol is obtained from the Poisson protocol by removing the intertrial interval, i.e., the recovery spike of the previous trial coincides with the first spike of the next trial (Fig. 8*A*). The single-sweep protocol removes the constraint of repeating the trials for putatively identical initial conditions.

We collected responses for the same recording time (∼2 min) with the three protocols described above. This corresponds to the same number of responses (on average) for the regular and the Poisson protocols (180 responses with the spike trains at 5 Hz), and, clearly, to a larger number of responses for the single-sweep protocol (440 responses on average, again with spike trains at 5 Hz). From the data so collected, we estimated the parameters of the synthetic connection with our method. The resulting distributions of relative errors (over 500 synthetic experiments) are shown in Figure 8*B*. Several points are noteworthy. First, the regular protocol appears to be the least effective one. Both the Poisson and the single-sweep protocol (Fig. 8*B*, red and purple box plots, respectively) led to estimates that are significantly more accurate than the estimates obtained with the regular protocol (blue box plots). Especially interesting is the fact that the Poisson protocol outperforms the regular one at parity of number of responses. This is because the trains of responses generated by the Poisson protocol are more variable, with the additional variability coming from the stochasticity of the presynaptic spike times. The stochasticity of the stimulation forces the synapse to visit a larger fraction of its possible hidden states and, consequently, to produce more informative sequences of observable responses (see below). Second, the single-sweep protocol outperforms the Poisson protocol. This is a consequence of the larger number of responses collected. At parity for the number of responses, the Poisson protocol produced more accurate estimates than the single-sweep estimate (data not shown). The reason is that, after a long intertrial interval (i.e., much longer than for *τ_D_* and *τ_F_*), the hidden state is known with very high probability. This, clearly, facilitates the estimation of the synaptic parameters. The increase in the number of responses collected during the same recording period led to more accurate estimates nonetheless.

The advantage of using the single-sweep protocol we have just illustrated does not depend on the specific parameters that were chosen for the synthetic connection or on the specific stimulation frequency. To show this, we calculated the FIM for randomly generated synthetic connections (as described in the Maximum-likelihood estimation versus least-squares fitting section) as a function of the stimulation protocol (i.e., regular, Poisson, and single sweep) and of the stimulation frequency. The FIM allows one to lower bound the variance that an unbiased estimator of the (continuous) synaptic parameters can achieve for a given protocol of stimulation. In other words, the FIM provides a precise measure of how informative the responses collected with the given protocol are about the parameters. The procedure followed to compute the FIM is described in Materials and Methods. Using the lower bound from the FIM, we computed the minimal relative errors achievable for all (continuous) synaptic parameters.

As an illustration, we plot in Figure 8*C* the cumulative distributions of these minimal relative errors for the facilitation time constant, for varying stimulation protocols and frequencies (recording time is 118 s). We chose to show the relative errors of the facilitation time constant because it is the parameter that is hardest to estimate, according to the FIM analysis. As can be seen, the single-sweep protocol (Fig. 8*C*, purple curves) outperforms the Poisson protocol (Fig. 8*C*, red curves) which, in turn, outperforms the regular protocol (Fig. 8*C*, blue curves) independent of the stimulation frequency. It is worth noting that the accuracy of the estimates is strongly dependent on the stimulation frequency for the regular protocol. On the other hand, this dependency is much weaker for both the Poisson and the single-sweep protocol. This can be intuitively understood by noticing that, with the regular protocol, the synaptic connection is probed only on two timescales (i.e., the interspike interval of the train and the recovery interval). If these two timescales are much longer than the synaptic dynamics timescales, these latter ones can only be poorly resolved. By contrast, the other protocols probe the dynamics with widely different interspike intervals (i.e., they are exponentially distributed).

## Discussion

We have introduced a new framework to quantitatively characterize patterns of transmission at chemical synapses in a statistically principled way. By explicitly modeling the sources of stochasticity present in the process, we obtained a parametric description of the probability of observing a sequence of postsynaptic responses upon a given pattern of presynaptic activation. The estimate is then obtained by selecting the parameters that maximize the likelihood of the observed synaptic responses. The uncertainty and the bias of the estimate are obtained by parametric bootstrap. If prior knowledge about the synaptic parameters is available, the whole procedure can be straightforwardly transformed into maximum a posteriori estimation. As one of the main sources of stochasticity stems from the quantal release of the neurotransmitter, our method naturally estimates the quantal parameters along with the dynamic ones from the same set of synaptic responses. Our method significantly outperforms the standard least-squares fitting procedure in terms of the accuracy of the estimates, irrespective of the available number of trials. This is because, unlike least-squares fitting, our method is able to extract information about the parameters contained in the correlation between synaptic responses and in their variability. When applied to experimental data, our method returns estimates of the parameters that are in excellent agreement with the published literature, despite the small number of synaptic responses (∼ 100) used and the larger number of parameters being estimated (Silver, 2003).

### Generality and robustness of the method

In this study, we have chosen the stochastic TM model as the underlying generative model (Fuhrmann et al., 2002). Several considerations motivated this choice: (1) the TM model has a small number of free parameters and yet is able to describe very diverse patterns of transmission; (2) The dataset we have analyzed was previously analyzed with the TM model, which allows for direct comparison; and (3) the stochastic TM model has been widely used in theoretical investigations of functional/computational implications of synaptic variability; thus, it appeared relevant to asses to which extent the model captures variability in real synapses. Our method, however, is general and can be extended to accommodate more biophysical detail, such as calcium-dependent docking rates (Dittman et al., 2000) or the presence of multiple vesicle pools (Rizzoli and Betz, 2005). Also, we emphasize that our method is not restricted to electrophysiological recordings and could be adapted to optical recordings (Yuste et al., 1999; Oertner et al., 2002; Trigo et al., 2012).

The frequencies used to probe synaptic transmission in our dataset were relatively low, and, accordingly, we have neglected postsynaptic receptor desensitization. However, desensitization is known to significantly contribute to short-term depression at higher stimulation frequencies (Trigo et al., 2012). This is especially relevant when experimentally probing synaptic connections with the Poisson protocol where interstimulus intervals can be very short. Our framework can be extended to include desensitization by making the quantal size dependent on spiking history. Explicit re-estimation formulas can be obtained and evaluated numerically in an efficient way also in this case (A. Barri and D.A. DiGregorio, unpublished observations).

Similarly, we have neglected heterogeneities across the release sites (Silver, 2003). Numerical experiments (data not shown) suggest that weak-to-moderate heterogeneities (coefficient of variation between 0.25 and 0.5) in the release site parameters are effectively absorbed in the estimate of the quantal noise. The estimates of the other parameters are very close to the averages of the corresponding distributions. The estimate of the number of release sites is unbiased.

### Comparison with previous studies

Few methods have already been proposed to estimate both quantal and dynamic parameters from the same set of responses. Saviane and Silver (2006) applied multiple-probability fluctuation analysis to the first response in the train to obtain the quantal parameters. Using these estimates, they subsequently extracted the release probabilities (modified by short-term plasticity) and quantal sizes (reduced by postsynaptic receptor desensitization) for each of the subsequent responses in the train. In a third step, the time constant of facilitation was extracted from paired-pulse ratios. Finally, the time constant of the docking process was estimated by fitting the TM model to the release probabilities for the different responses in the train (while keeping *τ_F_* fixed). Hallermann et al. (2010a,b) adopted a similar approach to compare the explanatory power of different models of repetitive transmission on the same dataset. Loebel et al. (2009) devised a somehow reversed procedure where, in a first step, least-squares fitting returns an estimate of the dynamic parameters, and of the product of the number of release sites and the quantal size (the so-called absolute synaptic efficacy). In the second step, another least-squares fitting returns the number of release sites (and thus the quantal size) that best reproduces the coefficient of variations of the synaptic responses.

All these methods proceed in a stepwise (and more or less laborious) manner using the estimates obtained in one step as fixed parameters in the next step. In this way, however, estimation errors in one step are propagated, and potentially amplified, in the next step. This should be of particular concern because the estimates in every step are obtained by least-squares fitting. All these methods are thus prone to the problems we have highlighted in the Maximum likelihood versus least-squares fit section, especially as the synaptic model is made more complex (i.e., features more parameters). Likewise, being based on least-squares fitting, all these methods require the repetition of the stimulation in identical conditions.

We are aware of only one other study (Costa et al., 2013) that has attempted to integrate the variability of the responses in the estimation of the synaptic parameters. There are, however, significant differences with our method. The method of Costa et al. (2013) does not take into account the correlation between different responses, which, as we have shown, carries a significant amount of information. Furthermore, Gaussian noise is assumed in the underlying generative model, which prevents one from resolving the quantal parameters. Importantly, the variance of the Gaussian noise has to be estimated from the experimental responses, which requires one to repeat the same stimulation protocols. Costa et al. (2013) also report that least-squares fitting of TM-like models can result in grossly inaccurate estimates of the parameters. This is interpreted as a consequence of a shallow and rugged error surface, which can be flattened and deepened around the true minimum by using Poisson instead of regular spike trains (consistent with previous suggestions from the studies by Sen et al., 1996, and Varela et al., 1997, and with our results). In contrast with this interpretation, we have shown that for a certain combination of parameters and regular spike trains the least-squares fitting procedure is ill posed.

### Advantages of the method

Our framework offers several advantages over existing state-of-the-art techniques for the characterization and quantification of synaptic transmission. The experimenter has complete freedom in choosing the stimulation protocol. For instance, the protocol can be chosen so as to elicit informative sequences of synaptic responses, thus achieving highly accurate estimates of the relevant parameters. As we have shown, for a given model, the asymptotic bounds on the accuracy of the estimates obtained with different protocols can be compared, before running any actual experiment, by computing the associated Fisher Information Matrices. Obviously, optimal protocols can be designed by using the same tool. Alternatively, and more interestingly, the stimulation protocol can be chosen so as to reproduce the statistical features of the *in vivo* spike trains driving the synaptic connections of interest. This would provide experimentalists as well as theoreticians with tools to develop effective descriptions of the transmission in physiologically relevant conditions. In fact, our method could already be fruitfully applied to investigate *in vivo* synaptic transmission at thalamocortical connections (Kaplan and Shapley, 1984; Ganmor et al., 2010).

To conclude, we would like to point out one potential application of our method that we deem especially interesting. Different mechanisms, both presynaptic and postsynaptic, have been proposed to be responsible for short-term synaptic plasticity (Zucker and Regehr, 2002; Fioravante and Regehr, 2011). It is presently unclear, however, whether these mechanisms are exclusive or, rather, they all cooperate to ensure the proper tuning of synaptic transmission across the wide range of possible patterns of presynaptic activity. Likelihood (once properly corrected for the differing number of free parameters) constitutes a theoretically principled metric to compare both the descriptive and predictive powers of different models over the same benchmark datasets. Our method, thus, paves the way to factorial testing of models of synaptic transmission, an approach that is increasingly and fruitfully being used in neuroscience and cognitive science (Pinto et al., 2009; Daunizeau et al., 2011; van den Berg et al., 2014).

## Synthesis

The decision was a result of the Reviewing Editor Gustavo Deco and the peer reviewers coming together and discussing their recommendations until a consensus was reached. A fact-based synthesis statement explaining their decision and outlining what is needed to prepare a revision is listed below. The following reviewers agreed to reveal their identity: Marco Beato, Mark van Rossum

This paper introduces a novel way of infer the parameters of synapse models with short term synaptic dynamics from empirical data using a machine learning method. Overall the paper seems correct and as it appears to present an improvement over most existing methods, it will well interest the community.

However, there is a number of weaknesses with the study that the authors should try to improve/explain:

1. Comparison with earlier methods (in particular the Loebel and Costa methods), apart from trivial Least Square fitting, is lacking.

2. Why was N not made part of the EM algorithm? Doesn't the method suffer from the step-wise manner they criticize at the bottom of p36.

3. Importantly, in contrast to the sampling method of Costa, this method does not yield uncertainty estimates in the parameters.

This certainly a disadvantage of the method, because strictly speaking a parameter by itself without uncertainty is useless.

The FIM could perhaps be used to approximate the uncertainties (although not as well as sampling, as it requires a Gaussian error landscape).

It would be worthwhile to examine if the FIM can be used for this purpose.

Related to that, I was surprised that in Fig7 both the errors across variables but also across protocols shows so little variation. Can the authors comment on this?

4. It is stated that the method extracts more information by considering more correlations, however, this is not shown directly.

If the statement is true, one would expect that extraction based on shuffled response trains should give different or less reliable parameters. Is that so?

In order to maximize utility for the community, I would certainly like to see the code be made public.

The method introduced by the authors obviously gives outstanding results for the types of synaptic contacts that were tested. However, I find that it would be more informative to test the procedure on a more extensive range of parameters and ideally up to its failure point. This would require benchmarking the procedure by using known sets of parameters to generate multiple synthetic experiments.

More specifically:

1) The "leave one out" procedure is correct to test for overfitting. However, once 500 synthetic experiments are generated from the same set of parameters (the ones defined on page 22, 8th line from bottom), it would be interesting to fit each of the 500 randomly generated experiments (different realization of the same model synapse) and show the actual distribution of the estimated parameters, so that one can compare the estimates to the real (known) values.

2) There is no question that the log-likelihood estimation is correct. However, the authors should give a measure of the confidence for each parameter in the likelihood space. More importantly, some parameters might be heavily correlated in the likelihood space. Since it is impossible to represent a 5-dimensional likelihood surface, I think the authors should plot the projection of the likelihood function two parameters at a time and more in general report the values of the correlation between all the parameters. The likelihood surface might be very steep in the direction of each parameter, but might take an elliptical shape if two or more parameters are heavily correlated. A typical example is that "apparently similar" experiments could be observed with high P, low N and high quantal variance, as well as with low P, high N and low quantal variance, just to mention the standard quantal parameters, without involving any short term plasticity time course.

3) I think it is really important to test the limit of applicability of the method for a range of different synaptic parameters. Ideally, I would like to see the distributions of estimated parameters over a number of synthetic experiments in which some critical parameters are varied.

a. One is the signal to noise ratio (for instance expressed as the ratio of the quantal size and the baseline noise), even though, given the deconvolution procedure used to estimate the peaks, I would not expect this to be too critical.

b. More importantly, can the procedure accommodate for large number of release sites? Ideally the parameters estimates for a number of synthetic experiments should be shown for a range of values of the total number of release sites (at least 100).

c. Similarly, different ranges of quantal variances should be tested, since large quantal variances, not uncommon at central synapse, could decrease the resolution of the procedure.

d. A range of initial Probabilities of release, going from very low to very high should be tested as well, since central synapses have ranges of P that vary widely between the extremes.

4) Throughout the paper, the quantal size Q is treated as a constant and there is only a brief mention to desensitization in the discussion (top of page 36). With Poisson trains at an average of 50 Hz (or more) the assumption of constant Q may fail and the short term depression will have both pre- and post-synaptic factors. This would not affect the performance of the procedure when using synthetic data, but it may invalidate the entire model in cases in which consecutive responses are affected by desensitization of post-synaptic receptors. The authors should discuss the feasibility of treating Q as a dynamic variable with a time index k as it is done for the probability of release.

5) Release sites are treated as identical and independent. There is little to argue about independence, but in many preparations the hypothesis of uniform release sites fails. The authors should at least comment on this.

Minor:

Furthermore Costa needed an extra parameter to describe some data. Is there a systematic deviation here as well?

It is stated that Costa et al achieved poor Least square fits because of the shallow and rugged error landscape.

I wonder if this is actually so different from the problem with least squares described here, or just a different way of saying the same.

Page 3 "Currently there is no method .." Both the Loebel and Costa method do.

I liked the included Table, but it would be good to note which area and from which animal the recordings were made, and made also at which temperature. This is a little bit of work but very valuable.

Also there are two entries for Bremaud for N,q and p0. What is the difference?

Why is the Costa analysis absent from this table?

The advantage of the Poisson protocol was already shown in Costa et al.

The single-sweep and Poisson protocol have the disadvantage that they don't allow to check non-stationarity.

A final repetition of a short segment would be useful in practice.

The Wang data was recorded with multiple frequencies, how was this accounted for?

Presentation issues:

- While overall the text is clear, there are a number of peculiar English constructions in the text. Proofreading by a native speaker could help.

- Fig2C has no x-axis label. Overall I did not think this representation to be very helpful.

The time development of the CV is hard to see. Why not plot CV + std dev vs time directly?

Fig3E no x-axis label.

Fig 4C. I would like to see the errors in the individual parameters, rather than this joint measure.

Fig 5B and 5C are anecdotal, which is fine, but as mentioned above, a proper analysis of the fitting results should also be included.

Fig 7B has no x-axis label

The labels in many figures are probably too small.

In some case it is not clear from the figure caption whether it is real or synthetic data.

Further minor points:

1) Please replace all occurrences of "to enlighten notation". The word "enlighten" has a different meaning. "algebraic cluttering" is what the authors want to reduce by not explicitly writing the dependence on the parameters set.

2) In equation 8, the set of parameters theta is not defined. Before the model is introduced explicitly, theta could be anything and the formalism is correct, but probably it would help the reader to mention explicitly the set of estimated parameters already at this stage

3) Figure 2C. Maybe better to use the number of connections in the y-axis of the histogram, rather than normalized frequency. Also, it would be probably more informative to split the experimental pairs into facilitating and depressing (either two separate sets of histograms or bars of different colours).

4) Table 1: it is reassuring to see that the numbers obtained by the authors are in line with what has been measured on most synapses. However, I find the information in the table not essential, because the data reported refer to several different preparations and synapses often recorded in very different conditions. It is out of doubt that the authors obtain perfectly plausible values, but the comparison with many of the listed papers is not significant.

5) I could not find any mention of how the code for analysing the data was written. The authors should state the programming environment used and where the code is/will be available.
